# Retrospective neutralization analysis of SARS-CoV-2 variants in early pandemic sera

**DOI:** 10.3389/fimmu.2026.1797240

**Published:** 2026-04-02

**Authors:** Mohamed Mahdi, Aya S. Al-Muffti, Tamás Richárd Linkner, Noémi Miltner, Olena Misák, István Várkonyi, József Tőzsér

**Affiliations:** 1 Department of Biochemistry and Molecular Biology, Faculty of Medicine, University of Debrecen, Debrecen, Hungary; 2 Department of Infectology, Faculty of Medicine, University of Debrecen, Debrecen, Hungary; 3 Doctoral School of Molecular Cellular and Immune Biology, University of Debrecen, Debrecen, Hungary

**Keywords:** COVID-19, immunity, neutralizing antibodies, pseudovirion neutralization assay, SARS-CoV-2 antibody

## Abstract

**Introduction:**

As the COVID-19 pandemic enters its sixth year, effective vaccination strategies remain a cornerstone, particularly given the limitations in access, timing, and efficacy of currently available antiviral therapies.

**Methods:**

In this retrospective study, we analyzed neutralizing antibody responses in serum samples from 100 vaccinated and boostered individuals using standardized cell-culture and in vitro neutralization assays. Samples were tested against the original Wuhan-Hu-1 spike protein as well as major SARS-CoV-2 variants of concern (B.1.351, B.1.617, and B.1.1.529/Omicron). We also investigated the potential for antibody-dependent enhancement (ADE) in monocyte-derived macrophages.

**Results:**

Only 61% of serum samples effectively neutralized the Wuhan-Hu-1 variant; among these, 20.6% demonstrated cross-neutralization of both B.1.351 and B.1.617. Of those cross-neutralizers, 66.6% were also able to neutralize Omicron. Notably, individuals who had been both vaccinated and previously infected showed stronger neutralizing responses than those who were only vaccinated and boostered. ADE was observed in 1% of samples.

**Discussion:**

This retrospective analysis offers a valuable insight to contextualize immune responses in real-world settings, revealing how actual immunological outcomes diverged from early expectations; at least in our studied population, and underscores the importance of continuously reassessing vaccine strategies as viral evolution unfolds.

## 1 Introduction

Coronavirus disease of 2019 (COVID-19) is caused by severe acute respiratory syndrome coronavirus 2 (SARS-CoV-2). Since the beginning of the pandemic in Wuhan China in 2019, as of the date of writing this manuscript, almost 779 million were infected and more than 7 million deaths have been reported worldwide according to the World Health Organization (WHO). COVID-19 is no longer considered a global emergency, but surges in cases are still being reported and continue to pose a significant risk to individuals with special health conditions (WHO).

Rapid viral evolution has led to the emergence of numerous SARS-CoV-2 variants of concern (VOCs) and sublineages with increased transmissibility and immune evasion capabilities (1, 2). As a result, many previously authorized monoclonal antibodies (mAbs); including bamlanivimab, casirivimab/imdevimab, and sotrovimab, have lost neutralizing activity against previous strains such as Omicron XBB.1.5, BA.2.86, and JN.1 (3–5). This has underscored the urgent need for broadly neutralizing antibodies (bnAbs) that target conserved viral epitopes (6, 7).

At the time of writing this manuscript, the WHO has granted Emergency Use Listing (EUL) to 12 COVID-19 vaccines (8). As of January 2026, around 70% of the global population has received at least one vaccine dose, with coverage significantly higher in high-income regions compared to low-income countries (9). These vaccines are categorized by platform: mRNA-based (Pfizer-BioNTech Comirnaty, Moderna Spikevax), non-replicating viral vector (Oxford-AstraZeneca Vaxzevria, Serum Institute of India Covishield, Janssen Jcovden, CanSino Convidecia), protein subunit (Novavax Nuvaxovid, Serum Institute of India COVOVAX, SK Bioscience SKYCovione), and inactivated virus (Sinopharm BIBP Covilo, Bharat Biotech Covaxin) (8). Except for inactivated vaccines, these platforms primarily target the SARS-CoV-2 S protein to elicit immunogenicity, with manufacturers regularly updating formulations to address emerging variants, such as the LP.8.1 strain (a JN.1-lineage Omicron subvariant) for the 2025–2026 season per FDA recommendations (10).

Variant-specific vaccines have shown improved neutralizing responses against circulating strains compared to earlier formulations. However, protection against infection remains partial and wanes over time, prompting the need for booster doses to maintain protection against severe disease (11).

Despite robust effectiveness in reducing severe disease and mortality, vaccine-induced immune responses vary across individuals due to host-related factors (12, 13). The effectiveness of vaccines vary depending on the vaccine type, the number of doses administered, and the circulating SARS-CoV-2 variant (14, 15), and while second vaccine doses and booster vaccinations significantly enhances protection against multiple SARS-CoV-2 variants (16), and provide protection against severe outcomes, the immunity is relatively short lived, waning significantly within 3–6 months after the second dose or booster, particularly against variants like Delta and Omicron (17, 18). However, immunity against severe outcomes is more durable, often lasting 6–12 months or longer, especially with boosters (19–21).

The decline in vaccine effectiveness over time is primarily attributed to waning neutralizing antibodies and antigenic drift in the S protein, particularly in Delta and Omicron variants (22–24). However, T cell–mediated immunity remains relatively preserved and plays a crucial role in preventing severe outcomes (25, 26). As such, combination vaccines have been explored to strengthen T cell responses (27–29).

While antibodies primarily target extracellular antigens, T cells recognize a broader range of epitopes, including those from intracellular proteins presented by infected cells via MHC molecules (30). The T cell-directed vaccine candidate BNT162b4, which encodes SARS-CoV-2 nucleocapsid, membrane, and ORF1ab proteins, has been shown to enhance T cell responses and reduce viral load and disease severity in hamster models when administered in combination with the BNT162b2 (Pfizer-BioNTech) vaccine (31). Immune response to SARS-CoV-2 infection or vaccination produces a diverse array of IgG antibodies targeting various regions of the nucleocapsid and the spike protein (32, 33). Mutations in the N-terminal domain (NTD) and receptor-binding domain (RBD) of Omicron variants have significantly diminished the efficacy of both mAbs and polyclonal sera from natural infection or vaccination (34–37).

While early studies reported high vaccine efficacy against infection (ranging from 61.7% to 99%) (38), such results must be interpreted cautiously. Many early studies did not adjust for confounding variables and were published prior to peer review, potentially overestimating real-world effectiveness. Meta-analyses indicate that vaccine effectiveness in preventing progression to severe disease ranges from 70–80% (17, 39, 40). However, effectiveness is reduced against Omicron and its subvariants, with noticeable waning 6 months after full vaccination (17, 39, 41).

Despite these limitations, vaccines remain associated with strong protection against hospitalization and death (42–44). Still, evolving viral characteristics and population behaviour, such as reduced testing or asymptomatic cases, may result in underreported infections and inflated vaccine efficacy estimates (45). Additionally, changes in testing practices and public health surveillance complicate data interpretation (44, 46, 47).

The antibody-dependent enhancement of infection (ADE) has been described in many viral infections; such as severe acute respiratory syndrome (SARS), and Middle East respiratory syndrome (MERS) coronavirus infections, as well as the respiratory syncytial virus (RSV) and dengue virus infections (48). While the mechanisms of ADE are variable, Fc receptors-mediated, and complement-mediated enhancement of infection, in addition to functional mimicry are the most commonly encountered phenomena (49, 50). Studies on ADE of SARS-CoV-2 infection are indeed lacking, and given the widespread use of vaccines in addition to booster vaccinations, characterization of vaccine-induced antibodies is of vital importance, especially in the phase of continuously emerging variants. Wang et.al., had identified ADE activity of SARS-CoV-2 neutralizing monoclonal antibodies utilizing SARS-CoV-2 pseudovirus infection in Raji and Daudi FcγRIIB expressing cells, effects of which were negated by introducing LALA mutation to the Fc region (51). Therefore, FcγRIIB, and not FcγRIIA or FcγRIA were determined to be involved in ADE of SARS-CoV-2 infection. This was further strengthened by the finding that primary B cells isolated from healthy human donors were susceptible to weak ADE of SARS-CoV-2 infection by these antibodies (52). To our knowledge, characterization and risk assessment of ADE of SARS-CoV-2 infection has not been thoroughly explored, especially in boostered individuals, and more so, in boostered individuals who had previously contracted the natural infection.

In this manuscript, we carried out an in-depth retrospective assessment on serum samples obtained from a cohort of 100 vaccinated and boostered health care workers (HCW) earlier on during the pandemic, in terms of their ability to neutralize SARS-CoV-2 prevalent variants utilizing single-cycle infection assays, and tested their susceptibility to ADE of SARS-CoV-2 pseudovirus infection.

## 2 Materials and methods

### 2.1 Sample collection

Blood was collected from individuals by venepuncture in serum collection tubes with gel separator during the period of October of 2020 to May of 2022. The samples were centrifuged at 2200 RPM for 10 minutes and the serum was transferred into Eppendorf tubes. The serum was then heat-inactivated by incubation at 56° C for 30 minutes, and thereafter stored at -70° C until use.

### 2.2 Cell lines and culture methods

HEK-293T and THP1 cell lines were all obtained from American Type Culture Collection (ATCC, Manassas, VA, United States), while ACE-2 and TMPRSS-2 expressing HEK293T (Hybrid-HEK293T) was purchased from GeneCopoeia (Rockville, MD, USA). Conventional HEK-293T cells were cultured in Dulbecco’s modified Eagle’s medium (DMEM) (Sigma-Aldrich, St. Louis, MO, United States) containing 10% FBS, 1% L-glutamine and 1% penicillin-streptomycin. Roswell Park Memorial Institute (RPMI) media (Sigma-Aldrich, St. Louis, MO, USA) was the media for THP-1 suspension cells supplemented with 10% FBS, 1% L-glutamine and 1% penicillin-streptomycin. Hybrid-HEK-293T were maintained in DMEM supplemented with 10% FBS, 1% L-Glutamine, 100 µg/ml Hygromycin and 1 µg/ml Puromycin. Passaging steps for HEK-293T were the following; media was removed and the cells were washed with Phosphate Buffer Saline (PBS) next, cells were detached from the surface of the flask with 0,25% (w/v) Trypsin-EDTA solution. Cells were then collected and centrifuged at 870 rpm, 24°C, for 5 minutes. The cells were then suspended into their respective media, and 0.5x10^6^ to 1.5x10^6^ cells were seeded back into the flasks. In the case of the Hybrid-HEK-293T, 3–5 hrs before passaging, the media was changed to DMEM containing 10% FBS and 1% L-glutamine to prepare the sensitive cells for trypsinization. Passaging steps for THP1 were the following; the cells were collected and centrifuged at 870 rpm, 24 °C, for 5 minutes. Next, the cells were re-suspended into RPMI media and 1-1.5x10^6^ cells were seeded back into a new flask. All cell lines were kept in a CO2 incubator at 37°C, 5% CO2, and 90-95% humidity. Cells were tested negative for mycoplasma before experiments.

### 2.3 Plasmids

For the production of SARS-CoV-2 S protein pseudotyped lentivirions, we utilized a second-generation lentiviral-based vector system. The following plasmids were used: pLenti (Addgene, MA, USA) as a GFP expressing transfer vector, psPAX2, as a packaging plasmid (a gift from Didier Trono from University of Geneva Medical School, Geneva, Switzerland). As to plasmids, encoding the envelope protein we used the following; pcDNA3.1-SPIKE encoding for S protein of SARS-CoV-2 (Genscript, NJ, USA); pLV-South African encoding for the B.1.531 variant of SPIKE (InvivoGen San Diego, CA, United States) and pcDNA3.1-SPIKE-Indian (Genscript, Piscataway, NJ, USA) encoding the B.1.617 variant of SARS-CoV2 S protein.

### 2.4 Production of SARS-CoV-2 S protein pseudotyped lentiviral virions

HEK-293T human embryonic kidney cells were used for the production of SARS-CoV-2 S-protein enveloped pseudovirions. pLenti, psPAX2, and pcDNA3.1-SPIKE or pLV-South African or pcDNA3.1-SPIKE-Indian plasmids were used in a 1:1:1 ratio as this ratio was found optimal for pseudovirion production. A day before transfection, HEK-293T cells were passaged to achieve a 70% confluence on the next day (3–5×10^6^ cells/flask). For all three types of pseudovirions, a total of 30 μg plasmid DNA was used for transfection using polyethyleneimine (PEI) (Sigma-Aldrich, St. Louis, MO, USA). PEI solution were added to the plasmid mixture and incubated for 20 minutes at room temperature. Next, the media was removed from the cells and replaced with 5 ml fresh DMEM media containing only 1% FBS without antibiotics. Plasmid mixture was then added to the media dropwise, and after the cells were incubated at 37 °C with 5% CO2 for 5h. The medium was then replaced with 8 mL DMEM containing 10% FBS, 1% penicillin-streptomycin and 1% glutamine. The supernatant was collected and filtered through a 0.22 μm polyvinylidene fluoride filter (Merck Millipore, Darmstadt, Germany) after 24 and 48 hours, respectively. The collected supernatants were then pooled together and concentrated by Amicon Ultra centrifugal filter units (Merck Millipore, Darmstadt, Germany). The concentrated viral particles were then stored at −70 °C. Enzyme-linked immunosorbent assay (ELISA) based colorimetric reverse transcriptase (RT) assay (Roche Applied Science, Mannheim, Germany) was used to determine the RT-activity of the pseudovirions according to the manufacturer’s instructions.

### 2.5 Differentiation of human monocyte-derived macrophages

THP-1 human monocytes were seeded into 48-well plate (35.000 cells/well) in 200 µl RPMI-1640 antibiotic-free culture medium for 3 hours. Then, the cells were activated by 50 nM phorbol-12-myristate-13-acetate (PMA) (Sigma-Aldrich, St. Louis, MO, USA) for 1 hour and PMA-containing medium then was removed. Thereafter, the cells were incubated in fresh 200 µl antibiotic-free culture medium for a further 24 hours to allow for differentiation into monocyte-derived macrophages (MDMs).

### 2.6 Neutralization assay and examination of ADE

HEK-293T hybrid cells were plated into 48-well plate (35.000 cells/well) in 200 µl selection media one day before experiment. Human serum samples were stored at -70 °C until use. On the day of the experiment, serum samples were thawed up and heat- inactivated for 30 minutes in 56 °C in dry block thermostat (Biosan, Warren, MI, USA). Then, we prepared a 10-fold dilution with free DMEM/RPMI from each serum samples and used 4 ng (RT- equivalent) of Wuhan-Hu-1, B.1.531 or B.1.617 S protein variant pseudovirions. The virus and diluted serum mixtures were incubated together; or pseudovirus and media as control, for 30 minutes at 37 °C. During the incubation time, the media was removed from the cells and fresh 100 µl from the appropriate media (selection media for HEK hybrid or culture RPMI for MDMs) was added to the cells. The mixtures were then added to the cells for 48 hours after which cells were resuspended in 400 μl cold PBS and the transduction efficacy was assessed by measuring percentage of GFP expression utilizing flow cytometry (FACS Calibur and FACS Aria, BD Biosciences, Singapore).

### 2.7 Antibody purification from serum and examination of ADE

Immunoglobulins were purified from a serum sample that showed potential ADE by affinity chromatography using ÄKTApurifier and a HiTrap^®^ Protein A High Performance affinity column (Cytiva Life Sciences, Wilmington, DE, USA). The column was equilibrated with 5–10 column volumes of binding buffer consisting of 20 mM sodium phosphate (pH 7.4). Serum samples were loaded onto the equilibrated column and the on-bound proteins were removed by washing the column with 10 column volumes of the same binding buffer until the absorbance returned to baseline. Bound antibodies were eluted using 0.1 M citric acid (pH 3.6). Elution fractions were collected into tubes containing 1 M Tris-HCl to immediately neutralize the low pH and prevent antibody denaturation. Fractions containing antibodies were identified based on absorbance at 280 nm and pooled. To remove the elution buffer and exchange the antibodies into a physiological buffer, pooled fractions were desalted using a HiTrap^®^ desalting column (Cytiva Life Sciences, Wilmington, DE, USA) according to the manufacturer’s instructions. The desalting column was equilibrated with 25 mM NaCl (pH 7.0), which was also used as the elution buffer. The pooled antibody solution was applied to the column, and the protein-containing fractions were collected.

Purified antibodies were quantified by measuring absorbance at 280 nm, yielding a concentration of 0.816 mg/mL. Purified antibody fractions were analyzed by SDS-PAGE under reducing conditions to verify their purity and approximate molecular weight. The antibodies were stored at -20 C until further analysis.

### 2.8 Human SARS-CoV-2 RBD enzyme-linked immunosorbent assay

For the quantification of SARS-CoV-2 receptor binding domain (RBD) of the S1 subunit of the spike (S) protein from SARS-CoV2 S protein pseudotyped lentiviral virions (Wuhan, B.1.531 and B.1.617 variants of S protein), we used Human SARS-CoV-2 RBD solid-phase sandwich ELISA kit (Invitrogen, Waltham, MA, USA) according to the manufacturer’s instructions. Absorbance was detected by Synergy H1 plate reader (BioTek, Winooski, VT, USA) at 450 nm.

### 2.9 Statistical analysis

Analysis and graph plotting was performed using GraphPad Software V.5 utilizing one-way ANOVA or unpaired-t test where applicable.

## 3 Results

### 3.1 Population and demographics

**Figure 1:**
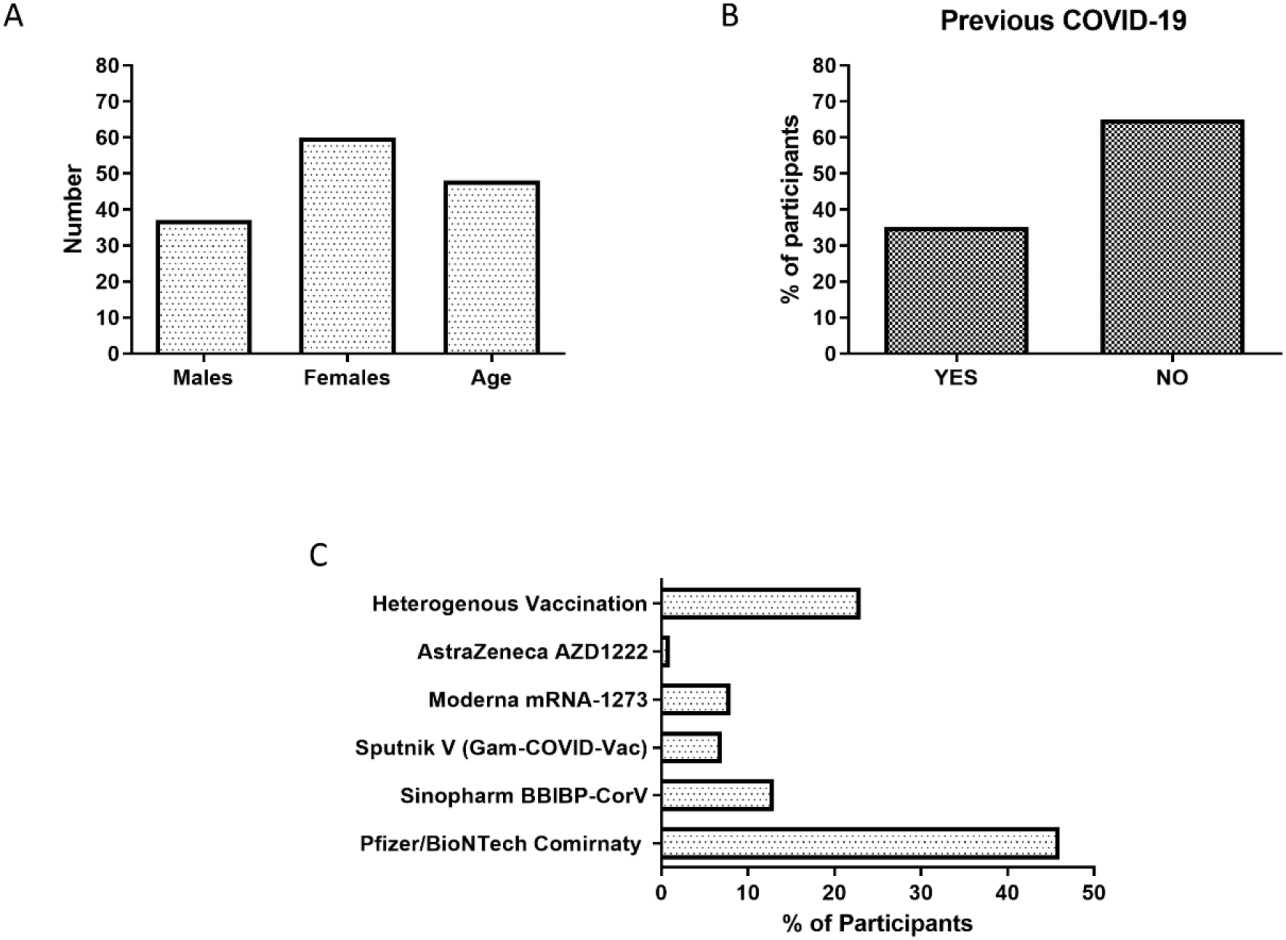
Participant demographics. **(A)** number of participants in terms of gender the average age. **(B)** Percentage of participants who had previously contracted COVID-19 and those who had not. **(C)** Type of vaccinations received plotted against percentage of the study population.

Our retrospective analysis was conducted on serum samples obtained from 100 HCWs and individuals attending our vaccination center at the Infectology Clinic, University of Debrecen Clinical Centre. An overview of participant’s demographics is highlighted in **Figure 1**. Age of the participants ranged from 22–89 years, and the average age was 48 years. Female participants were 59 (62%) and males constituted 36 (37.8%) of our cohort. Five individuals had not received previous vaccination against COVID-19, and their samples were utilized as controls. The majority of participants (54%) received mRNA-based vaccinations, 14% received inactivated vaccines, and 8% received vaccines based on non-replicating viral vectors. Average time from 2^nd^ vaccination shot to sample collection was 222.4 days (± 6.4). In the case of boostered individuals, average time from the receipt of the booster dose to sample collection was 70.6 days (± 10.6).

### 3.2 Cumulative assessment of neutralizing antibodies against the prototypical Wuhan-Hu-1

A pseudovirus neutralization assay utilizing the prototypical Wuhan-Hu-1 S protein was carried out on the one-hundred samples collected from HCWs and individuals attending our vaccination outpatient clinic, using 10x diluted serum samples. Samples that achieved 50% or more reduction of transduction were considered to be neutralizing, and were further used for analysis against the B.1.351 and B.1.617 pseudotyped S variants. Measurements were carried out in duplicates, and the average was calculated. The standard deviation within individual sample measurements averaged at 3.8%, 4.2%, and 5.8% in the case of the Wuhan-Hu-1 S, B.1.351 S, and B.1.617 S assays, respectively. Samples that neutralized all three variants, were tested against the B.1.1.529 (Omicron) variant in another *in vitro* assay.

**Figure 2:**
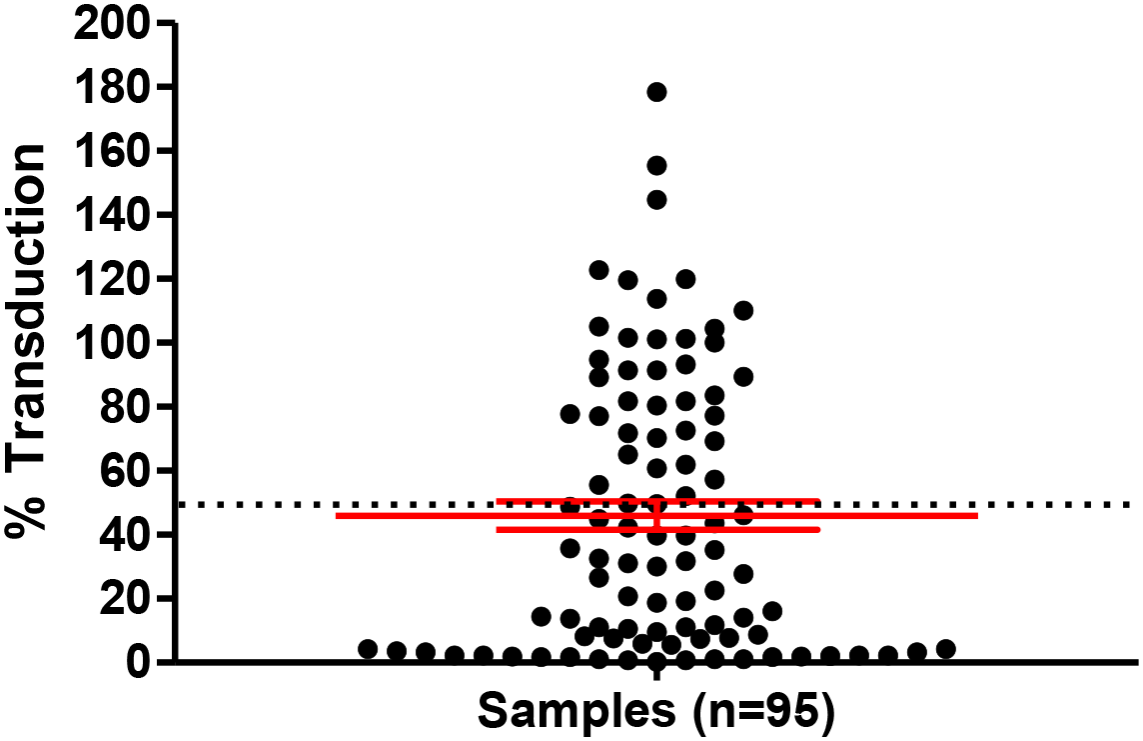
Cumulative assessment of serum samples. Pseudovirus neutralization assay utilizing lentivirions pseudotyped with the prototypical Wuhan-Hu-1 S protein. Results are representative of duplicate measurement. Percentage of transduction (single-cycle infection) is plotted on the Y axis. The mean and SE values are highlighted in red. Dotted line indicates 50% reduction of infection limit.

Altogether, 95 samples were included in the analysis, out of which 58 (61%) resulted in ≥ 50% reduction in infection by the Wuhan-Hu-1 S pseudotyped virions (**Figure 2**). The mean transduction capability of the pseudovirons in the presence of these samples was 45.9% (± 4.4).

### 3.3 Effect of previous infection on the development of neutralizing antibodies

**Figure 3:**
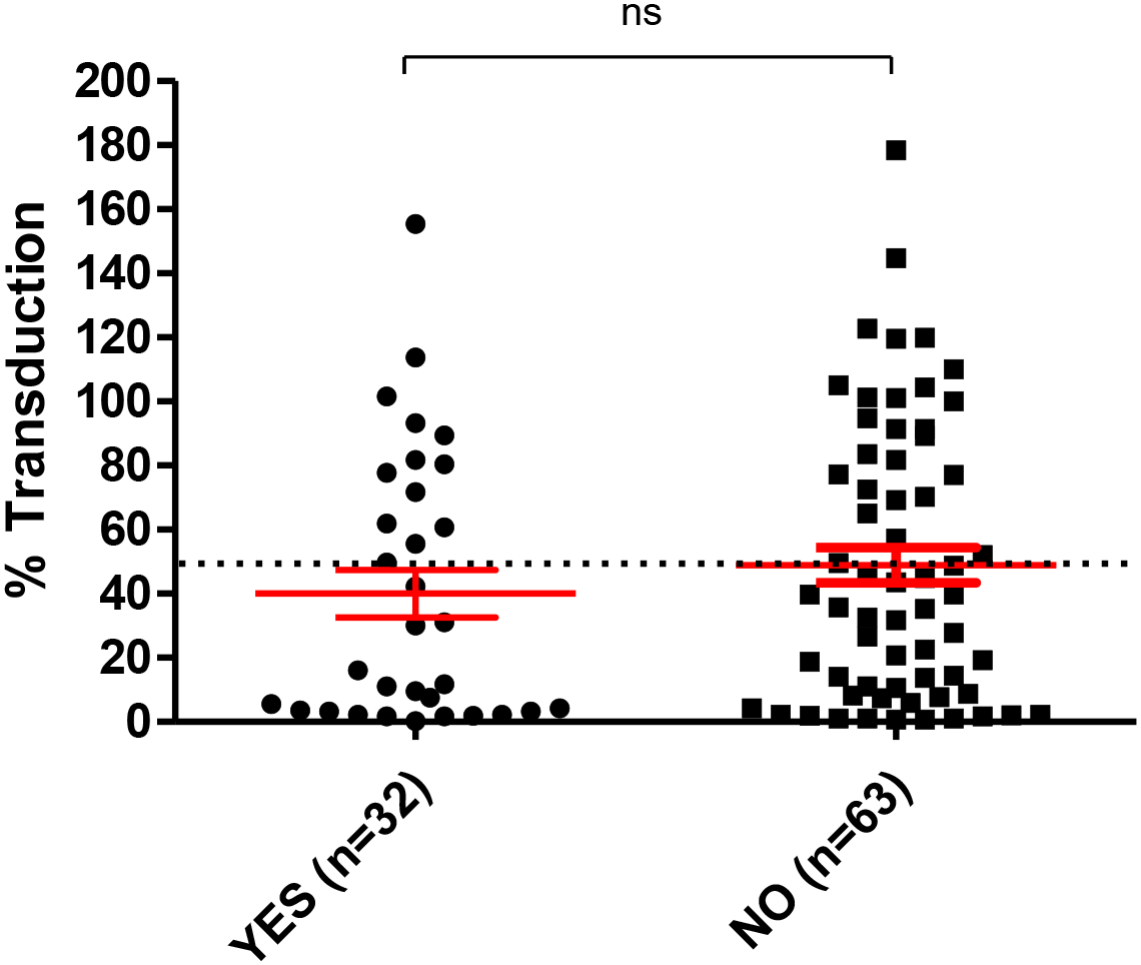
Stratification by previous infection status. Samples from 32 previously infected and 63 non-infected individuals were analysed in our neutralization assays utilizing lentivirions pseudotyped with the Wuhan-Hu-1 S protein. Percentage of transduction is plotted on the Y axis. The mean and SE are highlighted in red. Dotted line indicates 50% reduction of transduction limit. ns, non-significant, P value = 0.4.

In our study population, 32 individuals had documentation of previous SARS-CoV-2 infection by hospital records, while 63 reported no previous infection. In Wuhan-Hu-1 S-pseudotyped neutralization assays, there was no significant difference in neutralization of transduction between those who had previously encountered the infection and those who had not (**Figure 3**). Mean values of transduction were 40% (± 7.4), and 48.9% (± 5.4) for those who were seroconverted and those with no documented previous infection, respectively. It is worth noting that average time between documented infection and collection of serum sample was 9.7 months (standard error +/- 0.66).

### 3.4 Neutralization of other variants

**Figure 4:**
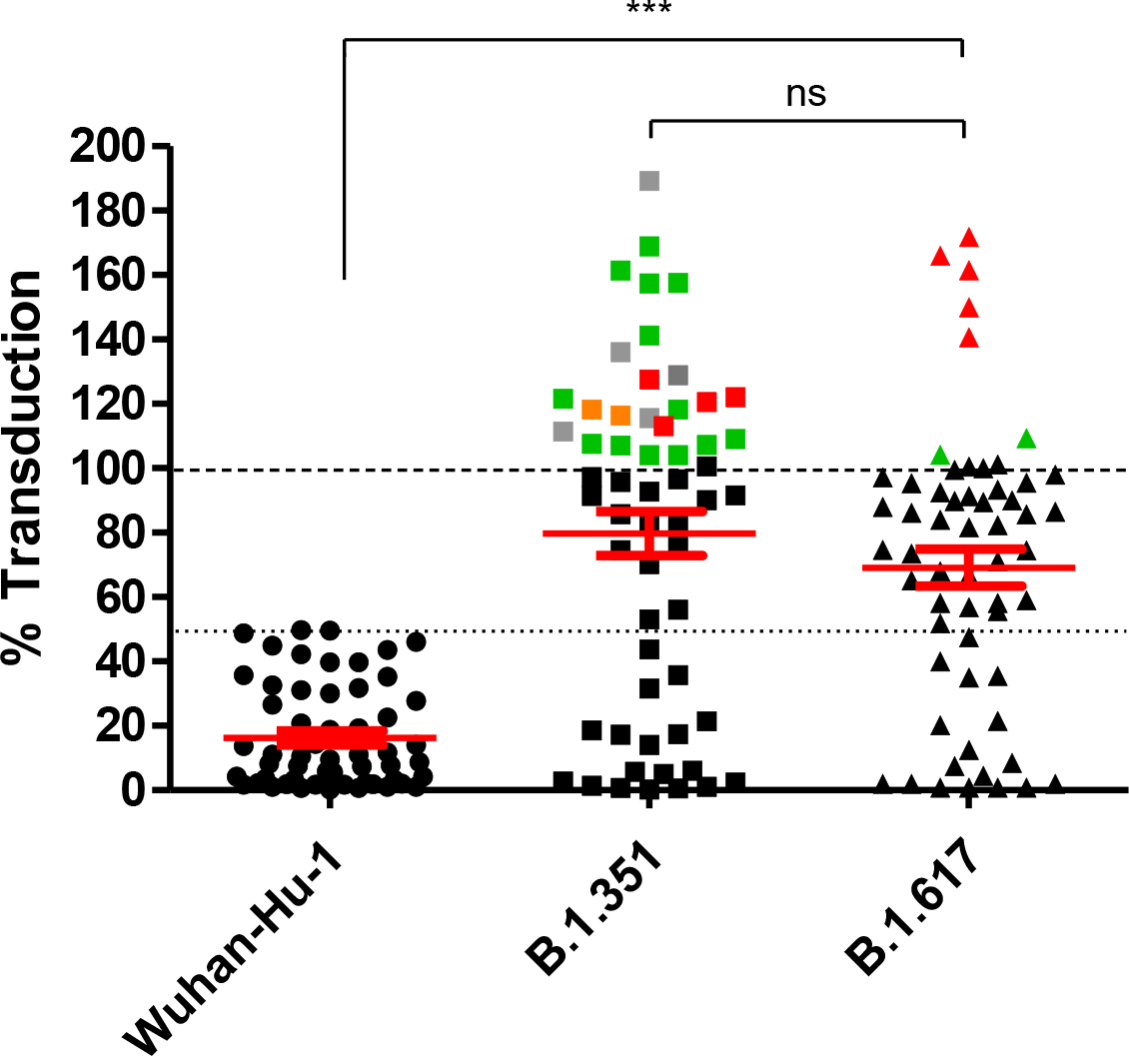
Neutralization of variants. Samples that showed ≥ 50% reduction in infection of the Wuhan-Hu-1 S pseudovirions were examined for cross-neutralization of other major variants. There was a significantly low cross-neutralization of the B.1.351 and 1.617 S pseudovirions. Statistical analysis was carried out by one-way ANOVA. Colour code: Gray: inactivated vaccine, Green: mRNA-based vaccine, Orange: Vector-based vaccine, and Red: heterologous vaccination. Percentage of infection is plotted on the Y axis. The mean and SE are highlighted in red. Dotted line indicates 50% reduction of infection limit. ns: non-significant, ***P value = < 0.0001.

Samples that resulted in ≥ 50% reduction of infection in the case of lentiviruses pseudotyped with the Wuhan-Hu-1 S protein were further analysed in neutralization assays utilizing the B.1.351 and B.1.617 S variants. Results showed that while 58 (61%) samples adequately neutralized the Wuhan-Hu-1 prototyped, only 18 (31%) and 15 (25.8%) of the 58 samples showed cross-neutralization of the B.1.351 and B.1.617 S pseudovirions, respectively (**Figure 4**). Mean values for neutralizations were 16.26% (± 2) for the Wuhan-Hu-1 S prototype, 80.6% (± 6.6) and 68.7% (± 5.5) for the B.1.351 and B.1.617 S pseudovirions, respectively. We have also noticed an enhancement of S variant mediated transduction in some samples, although, while this appeared to be associated with heterogeneous vaccinations in the case of B.1.617 S assays, a similar correlation could not be drawn in the case of the B.1.351 S variant.

Moreover, samples obtained that were able to neutralize all three variants adequately were tested further against the B.1.1.529 (Omicron) variant utilizing an *in vitro* SARS-CoV-2 surrogate virus neutralization assay (SARS-CoV-2 Surrogate Virus Neutralization test kit, GenScript, USA). Overall, eight samples out of the twelve (66.6%) showed positive neutralization of the Omicron variant.

### 3.5 Stratification of variant neutralization by vaccination status and previous infection

**Figure 5:**
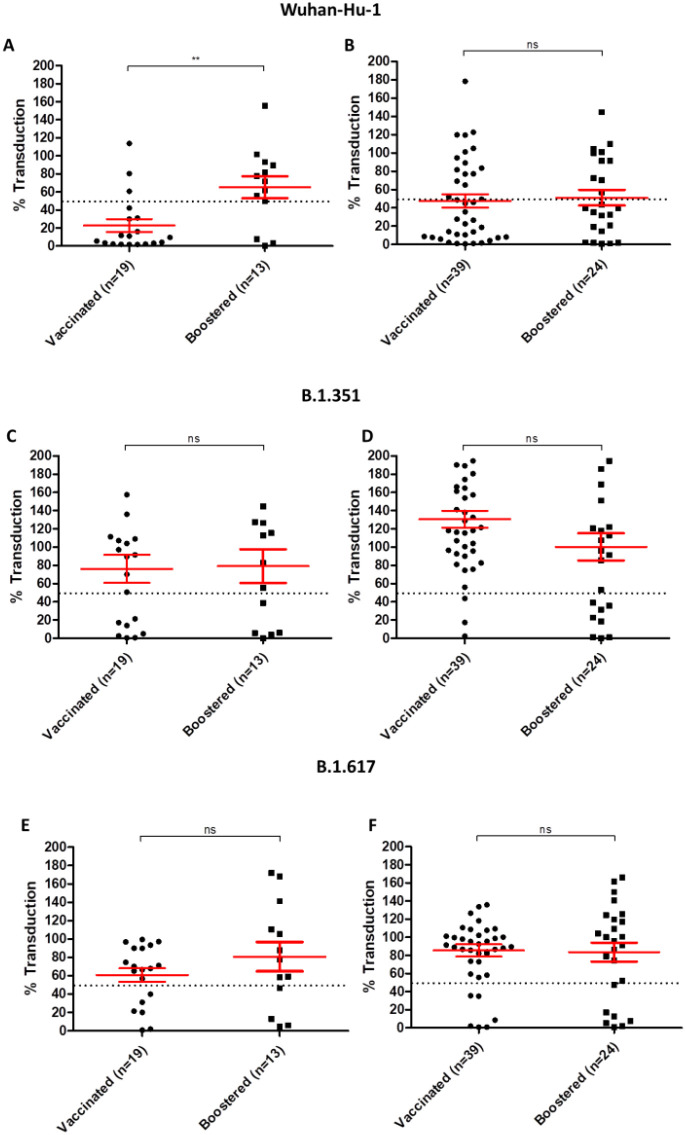
Neutralization of variants stratified by vaccination and previous infection status. **(A)** Neutralization assays utilizing the prototypical Wuhan-Hu-1 S in samples from individuals with documented previous infection, and **(B)** those with no previous history of infection. **(C)** neutralization assays using the B.1.351 S variant in previously infected individual samples and **(D)** samples with no previous infection. **(E)** neutralization assays using the B.1.617 S variant in previously infected, and **(F)** samples with no previous infection. The mean and SE are highlighted in red. Percentage of transduction is plotted on the Y axis. Dotted line indicates 50% reduction of infection limit. ns: non-significant, **P value = 0.003.

In a follow up analysis, we analysed neutralization of different SARS-CoV-2 S pseudovirions in fully vaccinated individuals compared to those who also received booster vaccinations. In case of the prototypical Wuhan-Hu-1 S, in individuals who had previously encountered the infection, neutralization of pseudovirions infection was more potent in the fully vaccinated group compared to those who also received booster vaccination (**Figure 5A**), while there was no statistically significant difference observed between the two groups in the case of those who had not previously contracted the infection (**Figure 5B**). On the other hand, no change in neutralization efficacy was observed in case of the B.1.351 variant in serum samples from previously infected individuals, nor in those who had not contracted the infection (**Figures 5C, D**). Finally, regarding the B1.617 variant, no difference was observed between the two groups, regardless of vaccination status (**Figures 5E, F**).

### 3.6 Exploration of antibody-dependent enhancement of SARS-CoV-2 entry

**Figure 6:**
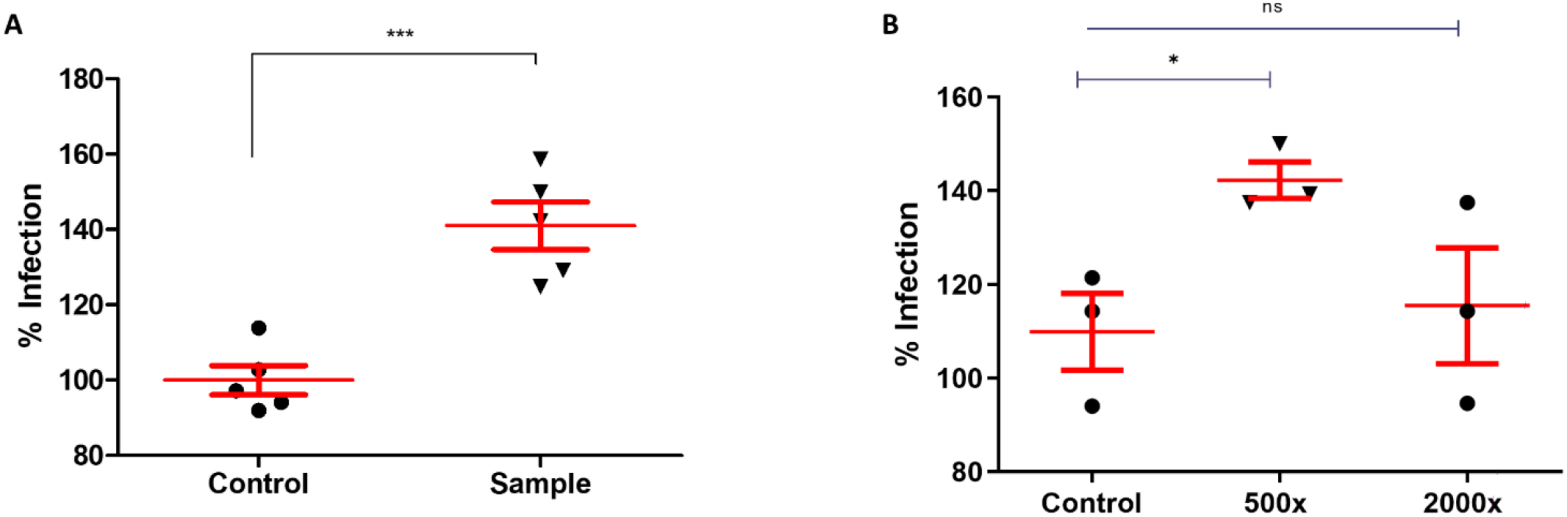
ADE of SARS-CoV-2 1.617 S pseudovirion infection. **(A)** Incubation of 10x diluted serum with 1.617 S pseudovirions resulted in enhancement of infection in activated THP-1 cells, compared to the control. The transduction percentage of the control was set as 100. Results shown are cumulative representation of 5 independent measurements. Percentage of infection is plotted on the Y axis, P value = 0.0006. **(B)** Transduction carried out in the presence of purified antibodies from the serum sample, using 500x and 2000x dilutions. Measurements were in triplicates. P value = 0.02, ns, non-significant. * indicates p value <0,05, *** pvalue <0,001.

To preliminary assess the development of antibody-dependent enhancement of SARS-CoV-2 entry in our samples, we choose samples that showed ≥ 150% increase of transduction efficiency compared to the controls; regardless of the variant assay type. Analysis was carried out on 18 samples in total, utilizing lentivirions pseudotyped with Wuhan-Hu-1, B.1.351 and B.1.617 S proteins in activated THP-1 cells in triplicate measurements. Out of all the samples analysed, a clear ADE of transduction was observed for one sample in the case of B.1.617 S variant pseudovirion, this was then confirmed by duplicate measurements and the cumulative results are represented in **Figure 6**. Moreover, antibodies were purified from this serum sample using affinity chromatography, and the transduction experiments were repeated with 500× and 2000× antibody dilutions. The results were consistent with those previously obtained using the unprocessed serum. This sample was obtained from HCW that had contracted COVID previously, fully vaccinated with an mRNA-based vaccine, and thereafter boostered with a single dose of an inactivated vaccine. To our knowledge, this individual had not been on immunomodulating drugs.

## 4 Discussion

In this study, we retrospectively analyzed the development of neutralizing antibodies in a vaccinated and boostered cohort of Hungarian healthcare workers and individuals attending a vaccination outpatient center, using samples collected during the early phase of the COVID-19 pandemic. We also attempted to explore the possibility of ADE following booster vaccinations, especially in those who had already contracted the infection previously. To carry out our experiments, we created SARS-CoV-2 S protein pseudovirions, utilizing the Wuhan-Hu-1, B.1.351, and B.1.617 S variants. Transduction efficiency of our produced pseudovirions was optimized, and we opted to achieve a transduction efficiency of around 20% for the variants, to avoid bias incurred by non-receptor mediated infection of target cells. The S protein expression in the utilized pseudovirions was also verified by Western-blot and ELISA, and the quantity adjusted for our assays.

Of the one hundred serum samples analysed, five were derived from individuals who were not vaccinated against COVID-19, nor had they previously contracted the infection according to the best of their knowledge, and hence, were utilized as controls for our experiments. Overall, 58 (61%) of the remaining samples showed adequate neutralization (≥ 50% reduction in infection) of the Wuhan-Hu-1 prototype, of which 18 (31%) were also able to neutralize the B.1.351 S variant, and 12 (20.6%) neutralized all three pseudovirions including the B.1.617 S variant. For those 12 samples, an *in vitro* surrogate virus neutralization assay was carried out utilizing the B.1.1.529 Omicron S variant, of which 8 (66.6%) showed positive neutralization.

When we stratified the results by status of previous infection, we were not able to detect a significant difference between the two groups, indicating that previous infection (probably with the Wuhan-Hu-1 virus) did not offer better protection against the infection, compared to the vaccinated, presumably seronegative group. This finding was expected, as the vaccines during that time were based on the original Wuhan-Hu-1 S sequence (53–55).

In order to assess the effect of booster vaccinations on the development of neutralizing antibodies, in regards to different variants, we stratified our findings by vaccination status and presence or absence of documented SARS-CoV-2 infection. Interestingly, booster vaccinations had resulted in a decrease of neutralization efficacy in those who had previously encountered SARS-CoV-2 infection, compared to only fully vaccinated individuals in case of the Wuhan-Hu-1 S type. While some studies have documented an increase in neutralizing antibody titer and memory B cell responses after booster vaccinations (56–58), others have voiced their concerns regarding booster vaccination in the absence of natural immunity acquired by SARS-CoV-2 infection, documenting profound impairment in type I interferon signaling by vaccination (59). A previous study found that antibody and memory B cell induction may be independent features of the immune response to mRNA vaccination (60), additionally, people with documented previous SARS-CoV-2 infection showed strong boosting of antibody and memory B cell responses after the first vaccine dose, with no additional rise in circulating antibodies, neutralizing titers, or antigen-specific memory B cells after the second dose (60). Moreover, immune exhaustion resulting from frequent exposure to vaccine-induced antigens is a phenomena that had been well documented, and cannot be ruled out in the case of vaccinations against SARS-CoV-2 (61, 62).

In regards to SARS-CoV-2 S variants, no change in neutralization efficacy was observed in case of the B.1.617 and B.1.351 S variants between the SARS-CoV-2 naïve individuals and those who previously encountered the infection, regardless of vaccination status.

Finally, we attempted to study the possibility of ADE in our cohort. During analysis of neutralization potential of samples, we came across samples that resulted in a significantly increased transducing potential of the pseudovirions which prompted us to explore the possibility of non-neutralizing antibody-mediated enhancement of infection in monocyte-derived macrophages. While previous studies had shown that sera of COVID-19 convalescent patients did not result in ADE of macrophage infection (63), others demonstrated FcγR-mediated ADE, and correlation between IgM and IgG levels and C1q-mediated ADE of SARS-CoV-2 infection (64). C1q‐mediated ADE had been well documented in HIV and other RNA viruses, promoting virus attachment and entry through interaction with the Fc portion of the antibodies and target cell surface receptors (65, 66). It is plausible that the enhancement of pseudovirion transduction observed for some samples in our ACE-II and TMPRSS2 expressing HEK-293T cell line was a result of similar mechanism, especially given the fact that HEK-293T cells are rich in C1q target receptors (67).

Although infection of monocytes and MDMs by SARS-CoV-2 was found to be abortive, the resulting cytokines secretion is thought to contribute to the pathogenesis of COVID-19 and the development of acute respiratory distress syndrome (ARDS) (68, 69).

In MDMs, ADE of pseudovirion entry was observed for one of the samples in the case of the B.617 S variant. This sample was obtained from a boostered HCW who had previously encountered the natural infection and had received heterogeneous vaccinations (mRNA-based complete vaccinations plus a single dose of inactivated vaccine as a booster). Given the lack of studies on ADE in SARS-CoV-2 infection, and the ever increasing rate of booster vaccinations compounded by the continuous emergence of new variants, this finding was indeed alarming, and prompts a thorough analysis and exploration of this phenomenon. Unfortunately, due to it being beyond the scope of our current study, we were not able to carry out detailed analysis of the antibodies involved regarding that sample, purification and analysis of which may provide important information, however, the mere finding itself, even though it only constituted 1% of the cases in our analysis warrants further investigation that we intend to explore in future studies.

Our study is not without limitations however. We relied on hospital data records and participant’s recollection to determine whether or not previous infection with SARS-CoV-2 had occurred. Unfortunately, some of the participants might have had previous asymptomatic or pausi-symptomatic infection which might have influenced our results. Additionally, level of antibodies produced through the course of natural infection, measured by ELISA-based methods were not determined by uniform standards, and many individuals participating did not have their antibody level determined, therefore, we opted to exclude this from our analysis to avoid bias. Although, the healthcare workers included in this study were subject to regular SARS-CoV-2 screening during the study period, in accordance with institutional policies implemented during the COVID-19 pandemic. No documented infections were recorded in these individuals prior to sample collection. While asymptomatic infections cannot be completely excluded, the likelihood of prior undocumented infection is considered low.

Moreover, the anti-SARS-CoV-2 vaccinations and booster vaccinations received were variable in our study population, with the majority of participants receiving mRNA-based vaccines. It is difficult to determine if the immunogenicity difference between the vaccines had a role in our study. Unfortunately, analysis of neutralization as a function of time since vaccination was not feasible in our cohort. The distribution of sampling times was strongly skewed toward later timepoints, limiting the ability to evaluate early post-vaccination neutralizing responses or to perform a statistically meaningful time-dependent analysis.

Finally, pseudovirus-based neutralization assays have their own set of limitations, of outmost importance being utilization of endocytotic pathways during infection, and perhaps utilization of accessory or alternative receptors, and hence, may not reflect the same results when compared to their natural virus counterpart.

Nevertheless, in this study, we have attempted to retrospectively explore the development of neutralizing antibodies in a cohort of Hungarian HCWs against major variants of SARS-CoV-2 S proteins, using comparable standards to previously published studies. While some findings were expected in our analysis, others were indeed of interest, and shall be of important use to clinicians dealing with the topic, especially given the fact that in the face of lacking mass-marketed potent antivirals, vaccination are expected to be the major players throughout this pandemic, that seemingly appears to be here to say for the long run.

Similar studies have analyzed variant cross-neutralization from using sera from HCWs (70). We believe our study provides important and complementary contributions. Our population of healthcare workers represents a highly exposed population with heterogeneous infection and vaccination histories, providing insight into real-world protective immunity rather than responses derived from narrowly defined or cross-sectional study populations.

Importantly, although the serum samples analyzed in this study were collected early in the COVID-19 pandemic, the data remain of significant scientific value. By utilizing a standardized cell culture-based neutralization assay, we were able to assess and directly compare the neutralizing antibody responses against several major SARS-CoV-2 variants, including the original Wuhan-Hu-1 strain, the Beta (South African) and Delta variants, as well as the early Omicron variant within the same cohort of samples obtained from healthcare workers. This standardized approach eliminates inter-assay variability and provides a unique longitudinal immunological snapshot of how early infection, or vaccine-induced antibodies interacted with evolving viral variants. Interestingly, 12 participants (20.6%) demonstrated neutralizing activity against all tested pseudoviruses. This subgroup may provide important insight into the characteristics of broadly protective antibody responses, and epitope analyses are currently underway to further characterize these antibodies.

Such retrospective analyses contribute to our understanding of immune escape, cross-neutralization, and the durability of immune responses, and serve as valuable references for future studies and vaccine development.

## Data Availability

The raw data supporting the conclusions of this article will be made available by the authors, without undue reservation.
